# Can the co-localization of CD8+, PD-1, PD-L1 AND PD-L2 patterns provide guidance in clinical evolution of patients with head and neck cutaneous squamous cell carcinoma?

**DOI:** 10.1016/j.bjorl.2025.101632

**Published:** 2025-05-24

**Authors:** Gustavo Nunes Bento, Andre Bandiera de Oliveira Santos, Leandro Luongo Matos, William C. Faquin, Sara I. Pai, Claudio Roberto Cernea

**Affiliations:** a Instituto do Câncer do Estado de São Paulo, Departamento de Cirurgia de Cabeça e Pescoço, São Paulo, SP, Brazil; b Faculdade de Medicina da Universidade de São Paulo, Departamento de Cirurgia (Disciplina de Cabeça e Pescoço), São Paulo, SP, Brazil; c Massachusetts General Hospital, Departments of Pathology, Surgery and Medicine, Boston, MA, United States; d Massachusetts General Hospital, Department of Surgery, Boston, MA, United States

**Keywords:** Cutaneous squamous cell carcinoma, Co-localization, PD-1, PD-L1, PD-L2

## Abstract

**Objective:**

Cutaneous Squamous Cell Carcinoma (cSCC) incidence has increased almost 200% in recent years. Blockade of immune checkpoint axes is an effective way to reactivate the host anti-tumor immune response across a variety of cancer types. We aimed to assess the presence of the Programmed cell Death-1 (PD-1):PD-L1/PD-L2 axis and associated CD8+ infiltration in head and neck cSCC, establish the co-localization criteria.

**Methods:**

This retrospective, cross-sectional study included 46samples of patients with high-risk cSCC. Multiplex staining for PD-1, PD-L1, PD-L2, and CD8 was performed, and images were captured by an epifluorescence microscope. 1%, 20% and 50% expression cutoff values were used, as well as the COX regression method to calculate the Hazard Ratio (HR) and 95% Confidence Interval (95% CI).

**Results:**

PD-1/PD-L1, PD-1/PD-L2 and PD-L1/PD-L2 co-localized in 80.4%, 45.7% and 47.8% of the patients, respectively. All patients exhibited CD8+ infiltration to some extent, with PD-1/PD-L1, PD-1/PD-L2 and PD-1/PD-L1/PD-L2 co-localized in 97.8%, 82.6% and 82.6% of the patients, respectively. There was no statistically significant relationship regarding the co-localization of immune checkpoint and the outcomes evaluated.

**Conclusion:**

This study has evidenced significant expression and co-localization of immune checkpoint axes in whole tissues and tumor infiltrate, emphasizing the potential application of co-localization as a valuable tool in the clinical management of cSCC. These findings provide important insights for understanding and optimizing therapeutic strategies in head and neck cSCC.

**Level of evidence:**

4.

## Highlights

• The co-localization of PD-1/PD-L1 could be a promising tool for prognostic evaluation.
• The first study to assess co-localization of CD8, PD1, PDL1, PDL2 in cSCC.
• Notable presence of CD8+T-cell infiltration in cSCC, co-localizing with PD-L1/L2.

## Introduction

Cutaneous Squamous Cell Carcinoma (cSCC) is a malignant neoplasm related to ultraviolet light exposure, scars, burns, immunosuppression, chemical agents and radiation.^1^, ^2^ Its incidence has increased almost 200% within the last three decades, with approximately 700,000 cases reported annually in the United States and more than 10,000,000 incident cases reported worldwide. Additionally, 60% of all cases occur within the head and neck region.^3^ While overall survival is above 90%, there are still up to 9,000 deaths attributed to cSCC annually worldwide.^4^

While surgical excision and adjuvant radiation are standard management for regional nodal disease, systemic chemotherapy and radiotherapy are commonly prescribed for metastatic disease.^5^ Treatment with immunotherapy is an attractive addition to systemic chemo-radiation, as UV-induced cancers are associated with a high mutation rate and, correspondingly, robust recruitment of inflammatory immune cells.^6^ The immune system plays a critical role in host defense against the development of cancer, including the development of local recurrence and/or metastatic disease. Tumor specific CD8^+^ T cells regulate their activities through the balance between co-stimulatory and inhibitory signals.^7^, ^8^, ^9^

Two of the most studied regulatory checkpoints are Cytotoxic T-Lymphocyte-Associated Antigen-4 (CTLA-4) and Programmed cell Death-1 (PD-1). CTLA-4 was the first checkpoint receptor targeted in cancer patients and it regulates the early stages of T-cell activation. CTLA-4 binds to B7-1 (CD80) and B7-2 (CD86) that are expressed on Antigen Presenting Cells (APCs). CTLA-4 is expressed on regulatory T-cells and is only upregulated in conventional T-cells after activation. When the receptor binds to its ligands, it downregulates T-cell immune responses. PD-1 belongs to the CD28 family and is expressed on cytotoxic CD8^+^ T-cells, regulatory T-cells, Dendritic Cells (DC), Natural Killer (NK) cells, macrophages, and B-cells.^10^, ^11^

The cognate ligands to PD-1 are PD-L1 (B7-H1) and PD-L2 (B7-DC). These are transmembrane proteins of the B7 superfamily, which also includes B7-1 (CD80), B7-2 (CD86), B7-H2, B7-H3, B7-H4 and B7-H6. PD-L1 and PD-L2 can both bind to PD-1 and CD80. PD-L1 can be expressed by T-cells, B-cells, myeloid dendritic cells and in tissue macrophages in the lung, kidney, liver, heart and placenta.^12^ PD-1/PD-L1 interaction regulates T-cell activities, modulating tolerance during pregnancy, autoimmune diseases, and malignant transformation. PD-L2 is expressed on APCs and some tumor cells, and the overexpression of PD-1 in the tumor environment can lead to a state of exhaustion among T-cells.^13^, ^14^, ^15^, ^16^, ^17^, ^18^

The expression of PD-L1 and/or PD-L2 proteins has been identified as a biomarker indicative of the response to PD-1 monoclonal antibody blockade. The relationship between prognosis and the response to blockers regarding the expression of immune checkpoints, as well as their concentration and localization in the inflammatory infiltrate habited by CD8+, remains to be elucidated. The comprehension of these findings may contribute to innovative approaches and therapeutic strategies, underscoring the significance of PD-L1 in the tumorigenesis process.

Based on reports of successfully targeting the PD-1/PD-L1 axis in head and neck squamous cell carcinoma, lung cancer, renal cancer and melanoma,^19^, ^20^, ^21^, ^22^, ^23^ we evaluated the relevance of targeting this pathway in cSCC by assessing PD-1, PD-L1, PD-L2 and CD8 expression in cSCC, as well as determining the co-localization of CD8^+^ PD-1 T-cells with ligands PD-L1 and PD-L2.

## Methods

### Patient clinical samples and demographics

Tumor specimens from patients diagnosed with head and neck cSCC between 2010 and 2016 at the Instituto do Câncer do Estado de São Paulo (Icesp), São Paulo, Brazil, were retrospectively identified. The study was independently reviewed and approved by the Sao Paulo Institute of Cancer (IRB 2.022526) and the Massachusetts General Hospital (IRB 2014P000559). Clinical and demographic data were extracted from the medical records.

Forty-six cases with skin or lip cancers were included in this study. Tumor adequacy was assessed by a head and neck pathologist (WCF). All patients were staged using the 8th Edition of the American Joint Committee on Cancer Cancer (AJCC) Staging Manual. Risk was classified according to National Comprehensive Cancer Network guidelines.^24^

### Multiplex immunofluorescence staining

Formalin-Fixed Paraffin-Embedded (FFPE) tissue sections were de-paraffinized in xylene for 10 min twice and dehydrated in graded ethanol solutions (2 × 100% ethanol for 5-min, 1 × 95% ethanol for 5 min, 1 × 90% ethanol for 5 min, 1 × 80% ethanol for 5 min, 1 × 70% ethanol for 5 min, and 2× deionized water) in preparation for multiplex Immunofluorescence (IF) staining. All slides were subjected to heat-induced epitope retrieval in Envision FLEX Target Retrieval Solution, pH 9.0, at 120 °C for 20 min and endogenous tissue peroxidases were quenched by incubating in 3% hydrogen peroxide solution for 7 min according to the manufacturer’s manual (DAKO, An Agilent Technologies, Santa Clara, CA).

The primary antibodies were diluted in an antibody diluent (DAKO, An Agilent Technologies, Santa Clara, CA) and incubated in a humidified chamber either for 1 h at room temperature or overnight at 4 °C, depending on the primary antibody applied. Antigen-antibody binding was visualized via application of the FLEX+Polymer system (DAKO, An Agilent Technologies, Santa Clara). The IF signal was amplified with Alexa Fluor 488, Alexa Fluor 555 and Alexa Fluor 647 Tyramide Super Boost kits (Invitrogen, Carlsbad, CA), or Alexa Fluor 750-conjugated anti-mouse IgG (Thermo Fisher Scientific, Waltham, MA). Fluorescence signal was preserved, and nuclei were visualized using Prolong Diamond Antifade Reagent with 4′,6-Diamidino-2-Phenylindole (DAPI, Invitrogen, Carlsbad, CA).

### Image capture and analysis

Fluorescence images were captured using a Zeiss 20X Plan-NeoFluar 0.5NA objective lens on the TissueFAXS whole slide scanning system (TissueGnostics, GmbH, Vienna, Austria) based on a Zeiss Axio Imager Z2 upright epifluorescence microscope. Analysis was performed using TissueQuest analysis software (TissueGnostics, Vienna, Austria). For analysis, cells were identified based on intensity-dependent segmentation of DAPI-stained nuclei. Analysis parameters were set to identify immunofluorescence for PD-1, PD-L1, PD-L2 and CD8 on a per-cell basis.

Immunofluorescence intensity was calculated for each cell from the average of pixel intensity for all pixels across the cell area. To determine specific and positive immunofluorescence, intensity thresholds were set for each channel based on non-specific control samples. Measurement of cell count mean fluorescence intensity, percentage of positive cells out of the total number of cells (% positive cells/all nucleated cells), and the density of positive cells were all calculated using the TissueQuest analysis software. Gates were created in intensity scatter plots for double-positive cells, and then compared across all marker combinations.

### Statistical analyses

Quantitative variables were calculated as mean and standard deviation, while qualitative variables were calculated as absolute and relative frequencies. Univariate analyses were done with the Cox regression method to calculate the Hazard Ratio (HR) and 95% Confidence Interval (95% CI). The statistical program SPSS® version 24.0 (SPSS® Inc; Illinois, USA) was used and in all comparisons, statistical significance level of less than 5% (*p* < 0.05) was adopted.

## Results

### Patient demographics

Thirty-five of the 46 (76%) patients included in the study were men and participant ages ranged from 48- to 96-years-old with a median age of 70-years. In our study, the most frequent primary site of tumor was the scalp 28.3% (13 out of 46), followed by preauricular 23.9% (11 out of 46), lip 15.2% (7 out of 46), malar 13% (6 out of 46), ear 6.5% (3 out of 46), others 13% (6 out of 46).

The average tumor thickness was 1.75 cm. All patients presented tumor thickness ≥0.2 cm and 56.5% of the cases (26 patients) had a tumor thickness ≥1 cm. In two patients, it was not possible to define total thickness due to bone invasion. In the Cox regression analysis, there was no relationship of tumor thickness to recurrence (*p* = 0.330) or death (*p* = 0.396). Nineteen of 46 (41.3%) patients had perineural invasion, but there was no statistical significance when stratifying risk of death (*p* = 0.583) or recurrence (*p* = 0.261) (Table 1).

**Table 1:** Clinicopathological characteristics and univariate analysis for recurrence and survival in patients with cutaneous squamous cell carcinoma in the head and neck region.

	PNI	Thickness > 1.0 cm	T3	T4	M1	Lymph node N1	Lymph node N2
No. (%)	19 (41.3)	26 (56.5)	17 (37.0)	3 (6.5)	2 (4.3)	4 (8.7)	5 (10.9)
*p*-value for Recurrence (HR)	0.26 (0.52)	0.33 (0.66)	0.93 (0.92)	0.07 (6.24)	0.03 (5.14)	0.71 (1.32)	0.55 (0.53)
*p-*value for Survival (HR)	0.58 (1.35)	0.40 (0.62)	0.49 (2.13)	0.04 (31.8)	0.04 (5.26)	0.92 (0.90)	0.58 (0.56)

HR, Hazard Ratio; M1, Metastasis stage 1; T3/T4, Tumor stage 3/4; N1/N2, Nodal stage 1/2; PNI, Perineural Invasion.

Locally advanced tumors (T3 and T4) were detected in 20 (43.5%) cases. The presence of regional lymph node metastasis occurred in 9 (19.6%) patients, and distant metastases in 2 (4.3%). Fifteen patients (32.6%) had locoregional recurrence, and 14 (30.4%) died due to disease related complications. In the Cox regression analysis, the presence of distant metastasis (*p* = 0.032) was an independent variable for predicting locoregional recurrence. For survival predictions, T4 tumors (*p* =  0.04) and distant metastasis (*p* = 0.039) were independent variables (Table 1).

### Follow up

Of the 46 patients, 15 (32.6%) had recurrence of the disease while 14 died (30.4%) in the period. Average follow-up time was 45.5 ± 27.6 months (1‒99). When patients who recurrence were analyzed, the mean time for the event was 37.9 months (median: 33 months; SD = 28.8 months). As for the occurrence of death, the average time for the event was 30.1 months (SD = 18.2 months).

### PD-1, PD-L1, PD-L2 and CD8 co-localization

**Fig. 1:**
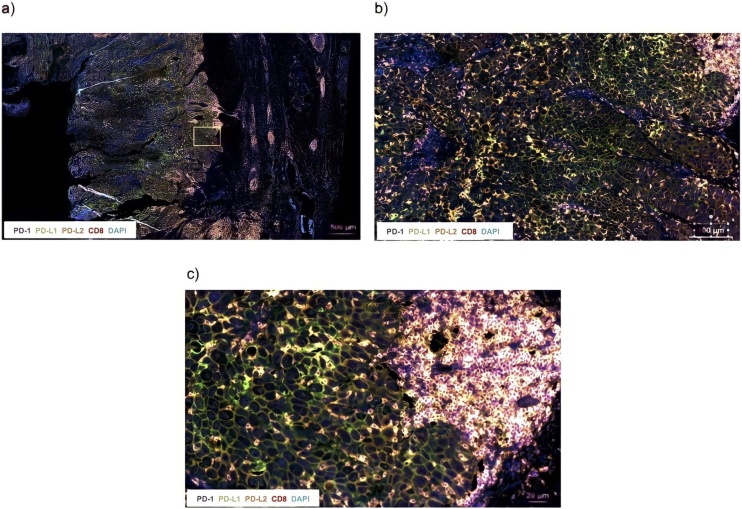
A representative example of multicolor immunofluorescence staining for PD-1 (Alexa Fluor 750, near-IR, membranous), PD-L1 (Alexa Fluor 488, green, membranous), PD-L2 (Alexa Fluor 555, orange, membranous), CD8 (Alexa Fluor 647, far-red, membranous) expression in head and neck cutaneous squamous cell carcinoma. DAPI (blue) was used to stain the nucleus. (a) Scale bar, 500 µm; (b) Scale bar, 100 µm and (c) Scale bar, 20 µm.

Samples were analyzed for PD-1, PD-L1, PD-L2, and CD8 expression. In addition, CD8 Tumor Infiltrating Lymphocytes (TILs) were analyzed for co-expression of PD-1, PD-L1 and PD-L2. PD-L1 and PD-L2 positivity was scored using three cut-offs: >1%, >20% and >50% (Fig. 1).

Next, we determined the co-localization of PD-1/PD-L1: 80.4% (37 of 46) of the tumor samples had co-localization in >1% of the cells, 4.3% (2 of46) co-localized in > 20% of the cells, and none co-localized in > 50% of the cells. For PD-1/PD-L2 interaction, 45.7% (21 of 46) of the patients co-localized in > 1% of the cells, 2.2% (1 of 46) co-localized in >20% of the cells, and none co-localized in > 50% of the cells. PD-L1 and PD-L2 co-localized in >1% of cells in 47.8% (22 of 46) of patients, while they co-localized in >20% of cells in 4.3% (2 of 46) of patients. No patients had PD-L1/PD-L2 co-localization in >50% of the cells (Table 2).

**Table 2:** Cut-off points of checkpoints expressions and presence on whole tissue samples of head and neck cutaneous squamous cell carcinoma.

Checkpoints	Percentage (%)	Samples No. (%)
PD-1	>1%	37 (80.4)
	>20%	2 (4.3)
	>50%	0 (0)
PD-L1	>1%	39 (84.8)
	>20%	6 (13.0)
	>50%	1 (2.2)
PDL-2	>1%	22 (47.8)
	>20%	2 (4.3)
	>50%	0 (0)
CD8	>1%	40 (87)
	>20%	2 (4.3)
	>50%	0 (0)
PD-1/PD-L1	>1%	37 (80.4)
	>20%	2 (4.3)
	>50%	0 (0)
PD-1/PD-L2	>1%	21 (45.7)
	>20%	1 (2.2)
	>50%	0 (0)
PD-L1/PD-L2	>1%	22 (47.8)
	>20%	2 (4.3)
	>50%	0 (0)

CD8, Cytotoxic T-lymphocytes; PD-1, Programmed cell Death 1; PD-L1/2, Programmed cell Death ligands1 and 2.

When we focused the analysis on the CD8^+^ T-cells, 100% of the patients had PD-1 expression in >1% of the cells, 80.4% (37 of 46) of patients had >20% of cells expressing PD-1, and 47.8% (22 of 46) of patients had expression in >50% of the cells. PD-1/PD-L1 interaction in >1% of the cells occurred in 97.8% (45 of 46) of the patients, while 78.3% (36 of 46) of the samples demonstrated PD-1/PD-L1 co-localization in >20% of the cells and 37% (17 of 46) of the cases also showed PD-1/PD-L1 co-localization in >50% of cells. PD-1/PD-L2 co-localized in >1% of the cells in 82.6% (38 of 46) of samples, while 43.5% (20 of 46) of samples showed co-localization in >20% of the cells and 21.7% (10 of46) demonstrated co-localization in >50% of the cells. We found PD-1/PD-L1/PD-L2 co-localized in >1% of the cells in 82.6% (38 of 46) of the patients, while 41.3% (19 of 46) of the patients showed expression in >20% of the cells, and 19.6% (9 of 46) of the patients simultaneously expressed all three checkpoints in >50% of the cells (Table 3).

**Table 3:** Cut-off points of checkpoints expressions and presence on Tumor Infiltrating Lymphocytes (TIL) of head and neck cutaneous squamous cell carcinoma.

Checkpoints on TIL	Percentage (%)	Samples No. (%)
PD-1	>1%	46 (100)
	>20%	37 (80.4)
	>50%	22 (47.8)
PD-1/PD-L1	>1%	45 (97.8)
	>20%	36 (78.3)
	>50%	17 (37)
PD-1/PD-L2	>1%	38 (82.6)
	>20%	20 (43.5)
	>50%	10 (21.7)
	>1%	38 (82.6)
PD-1/PD-L1/PD-L2	>20%	19 (41.3)
	>50%	9 (19.6)

PD-1, Programmed cell Death 1; PD-L1/2, Programmed cell Death ligands 1 and 2; TIL, Tumor Infiltrating Lymphocyte.

To establish a connection between immunoreceptors and the clinical outcomes of patients, defined as recurrence or overall survival, the Cox regression method was applied to those cutoff points established as >1%, >20%, and >50%, as well as the cutoff points established by the ROC curve. The optimal cutoff point was calculated for all immune checkpoints and their co-localizations, both in the total tissue sample and in areas populated by cytotoxic T lymphocytes (CD8+).

**Fig. 2:**
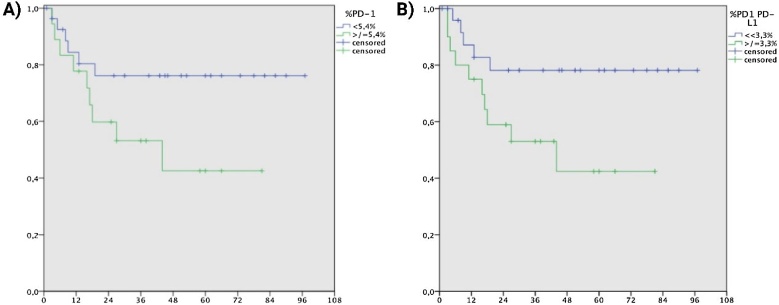
(A) Kaplan-Meier Curve: PD-1 expression ≥5.426% (immunofluorescence) in a series of operatively treated cutaneous squamous cell carcinomas between 2010 and 2016 and recurrence occurrence during follow-up (*p* = 0.091). (B) Kaplan-Meier Curve: PD-1/PD-L1 expression ≥3.345% (immunofluorescence) in a series of operatively treated cutaneous squamous cell carcinomas between 2010 and 2016 and recurrence occurrence during follow-up (*p* = 0.054).

When defining the odds of recurrence and overall survival, no significant values (<0.05) were found for any of the variables. The most promising variables are PD-1 ≥5.426% (*p* = 0.091; RR = 2.444; 95% CI between 0.867 and 6.893) and the co-localization PD-1/PD-L1 ≥3.345% (*p* = 0.054; RR = 2.892; 95% CI between 0.983 and 8.505) for the entire tissue, both associated with the risk of recurrence (Fig. 2).

When evaluating TIL (tumor-infiltrating lymphocytes), statistical relevance was also not found in the relationship with recurrence or overall survival. The most promising results identified in attempting to correlate the expression of immunoreceptors and their co-localizations were PD-1 ≥ 49.32% (*p* = 0.138; RR = 1.040; 95% CI between 0.365 and 2.967) and the co-localization PD-1/PD-L2 ≥ 8.974% (*p* = 0.138; RR = 2.606; 95% CI between 0.735 and 9.242) to define the odds of recurrence.

## Discussion

We demonstrate that the expression of immune checkpoints in cSCC is high, especially within the immune-rich tumor microenvironment. To our knowledge, this is the first study to quantify the co-localization of PD-1, PD-L1, PD-L2, and CD8 in cSCC. The FDA has recently approved the use of anti-PD-1 cemiplimab for advanced unresectable or metastatic cSCC based on its ability to block interactions between PD-1 and its ligands PD-L1 and PD-L2.^25^ In the initial phase 1 study, response to cemiplimab was observed in 50% (13 of 26) of patients, which was nearly identical to the 48% (28 of 59) response rate in the phase 2 metastatic-disease cohort.^25^ Based on the results of the KEYNOTE-629 trial, the FDA has also expanded the prescribing guidelines of pembrolizumab to cover patients with recurrent or metastatic cSCC that is not curable by surgery or radiation.^26^ After treatment with pembrolizumab, the objective response rate was 34%. Despite the promising results of cemiplimab and pembrolizumab use in treating patients with advanced unresectable or metastatic cSCC, the role of the PD-1:PD-L1/PD-L2 axis in cSCC had not been well elucidated.

PD-L1 and PD-L2 are also expressed on several cells. The expression of PD-L1 is more abundant compared to PD-L2, and his presence is also correlated with the PD-L2, both in the overall tissue and specifically in areas inhabited by CD8+ cells. PD-L1 is expressed in dendritic cells, lymphocytes, and macrophages, and also by pancreas, spleen, liver, eye, vessel and placental cells.^12^ PD-L2 is expressed in antigen-presenting dendritic cells, macrophages, lymphocytes and bone marrow cells.^12^ PD-L1 shows greater expression in the thymus cortex, whereas PD-L2 is more expressed in bone marrow. Both are stimulated by IL-2, IL-4, IL-7, IL-10, IL-15, IL-21, and IFN-γ. PD-1 signaling within lymphocytes inhibits the activity of CD28 and antigen presentation to inhibit cell proliferation and decrease cytotoxic activity.^14^, ^15^, ^16^, ^17^

Many solid tumors have increased expression of PD-L1 that can dampen anti-tumor immune responses. While previous studies have evaluated the role of PD-L1 in high risk skin squamous cell carcinomas, none have described their colocalization with CD8^+^ T-cells in high risk cSCC.^27^, ^28^, ^29^, ^30^ In this study, a higher chance of recurrence was demonstrated in patients who exhibited co-localization of PD-1/PD-L1 > 3.3%, although this value did not reach statistical significance; nevertheless, we observed a statistical trend. Our work is the first assessing both the frequency of expression and co-localization of the receptors and ligands.

Studies have been made to quantify the expressions of these immunoreceptors, as well as to establish a relationship between them and the types of inflammatory cells present in the tumor microenvironment. In a study with 74 patients from Australia, PD-L1 > 1% was present in 52.7% of the samples. When PD-L1 was >5%, 70% of patients demonstrated robust inflammatory infiltration and improved prognosis that may be explained by an inflammatory phenotype common to cSCC.^19^ Because cSCC is primarily derived from UV radiation, cSCCs have a high tumor mutation burden that can elicit a host anti-tumor immune response, as evidenced by the presence of CD8^+^ lymphocyte infiltration in all cases in our study.^31^, ^32^, ^33^ Patients with tumors that have high mutation burdens are also more likely to respond to immunotherapy with a checkpoint inhibitor, presumably because the tumors have greater neoantigen expression.^31^, ^32^, ^33^

Another study characterized the expression of PD-L1 in tumor cells with advanced cSCC, finding its presence in 41% of the patients, a strong relation between inflammation and PD-L1 expression, and the probability of lymph node metastasis when expression was identified on more than 25% of the cells in the tumor microenvironment.^29^ Jiao et al. found the presence of PD-L1 and PD-L2 on 40% and 60% of cells, respectively, in 61 cases of cSCC. While CD83^+^ was found on 39% of cells, it was detected on 37% of PD-L1 positive samples and on 33% of PD-L2 positive samples. Meanwhile, PD-L1 and PD-L2 were detected on 39% and 51% of CD83^+^ positive samples, respectively. Co-localization of PD-L1, PD-L2 and CD11c^+^ dendritic cells has also been described.^34^

Varying techniques for assessing PD-1, PD-L1, and PD-L2 expression, either by cellular location (cell membrane or cytoplasm), reading form (manual or automatic), or by different immunofluorescence techniques bring different results in the literature and result in challenges for establishing positivity, degree of expression, and correlations with prognosis and response to immunotherapy. The cut-off point for positivity varies considerably for PD-1, PD-L1, and PD-L2 based on the antibody used, ranging from any positivity (> 1%) to 5%, 10%, 15%, 20%, 25%, or even 50%.^11^, ^15^, ^17^, ^18^^,^^29^, ^35^, ^36^, ^37^, ^38^

Our study suggests that the presence of PD-1 and PD-L1 and its interaction may play a role in the progression of cSCC of the head and neck region. In addition, there was a significant detection of CD8 and PD-L2 expression in the tumor microenvironment, paving the way for further studies in an attempt to fully understand the immune microenvironment of advanced cSCC.

## Conclusion

In summary, we identified a significant infiltration of robust CD8 + T-cells in cSCC, which co-localized with PD-L1 and PD-L2. This reaffirms the importance of PD-L1 presence in the pathogenesis tumoral. It was not possible to establish a relationship between the expression of immunoreceptors and/or their co-localizations and clinical outcomes; however, a worse clinical outcome was observed in patients with PD-1/PD-L1 co-localization > 3.3%. Moreover, these findings suggest the potential for guiding personalized clinical management in patients with advanced tumors, predicting treatment response, and clinical disease progression. Our results may stimulate the development of further combinatorial studies to enhance clinical responses to immunotherapy.

## Funding

This work was supported by the Universidade de São Paulo.

## Declaration of competing interest

The authors declare no conflicts of interest.
